# Recommendations of physical activity and rest in a Colombian prenatal control program

**DOI:** 10.11606/S1518-8787.2019053000934

**Published:** 2019-04-26

**Authors:** Myriam Ruiz-Rodríguez, Yuri Sánchez-Martínez, Paula Camila Ramírez-Muñoz, Diana Marina Camargo-Lemos

**Affiliations:** I Universidad Industrial de Santander. Departamento de Salud Pública. Bucaramanga, Colombia; II Universidad Industrial de Santander. Escuela de Fisioterapia. Bucaramanga, Colombia

**Keywords:** Pregnant Women, Exercise, Rest, Prenatal Care, Mujeres Embarazadas, Ejercicio, Descanso, Atención Prenatal

## Abstract

**OBJECTIVE:**

To determine the frequency of the registry of physical activity and rest recommendations made to pregnant women and to explore their associated factors in a prenatal care program of primary care public institutions in Bucaramanga, Colombia.

**METHODS:**

An observational study was conducted. The sampling frame consisted of the medical records of the pregnant women who attended at least one prenatal care program between January 1 and December 31, 2012 (n = 2.932), in 21 primary care health centers. We analyzed sociodemographic variables, prenatal and clinical antecedents, and information related to health personnel and the organization of health centers as possible factors associated with the recommendations of physical activity and rest recorded in the clinical history. Logistic regression models were applied to explore associations with α = 0.10.

**RESULTS:**

There was a frequency of 26.1% of PA recommendations and 3.6% of rest recommendation on record, issued by nutrition (97.3%) and medical (86.7%) professionals, respectively. The factors associated with the registration of physical activity recommendations were: being nulliparous pregnant (OR = 1.7), attending more than four Prenatal Care Attention Programs (OR = 2.2), having high or medium obstetric risk in the first prenatal care program (OR = 0.6), and being attended in the western (OR = 0.5) and eastern (OR = 0.2) administrative areas health centers.

**CONCLUSIONS:**

The low frequency of physical activity recommendations found in the records makes it necessary to reinforce the management strategies of health centers and strengthen the monitoring and accompaniment to comply with the care protocols. In addition, it is necessary to train health teams on the benefits of physical activity and their proper prescription, considering the multiple benefits derived from their practice on the maternal-fetal health.

## RESUMEN

**OBJETIVO:**

Determinar la frecuencia del registro de recomendaciones de actividad física y reposo realizadas a las gestantes y explorar sus factores asociados, en un programa de atención de control prenatal de instituciones públicas de primer nivel de atención en Bucaramanga, Colombia.

**MÉTODOS:**

Se realizó un estudio observacional. El marco muestral estuvo conformado por los registros médicos de las gestantes que asistieron al menos a un programa de atención de control prenatal entre el 1º de enero y 31 de diciembre de 2012 (n = 2,932), en 21 centros de salud de primer nivel de atención. Se analizaron variables sociodemográficas, antecedentes prenatales, antecedentes clínicos, e información relacionada con el personal de salud y la organización de los centros de salud como posibles factores asociados con las recomendaciones de actividad física y reposo registrados en la historia clínica. Se aplicaron modelos de regresión logística para explorar las asociaciones con un α = 0.10.

**RESULTADOS:**

Se observó una frecuencia de 26.1% de registro de recomendaciones de AF y 3.6% de reposo, emitidas por parte de profesionales de nutrición (97.3%) y medicina (86.7%), respectivamente. Los factores asociados al registro de las recomendaciones de actividad física fueron: ser gestante nulípara (OR = 1.7), asistir a más de cuatro Programas de Atención de Control Prenatal (OR = 2.2), tener riesgo obstétrico alto o medio en el primer programa de atención de control prenatal (OR = 0.6) y ser atendidas en los centros de salud de las zonas administrativas de occidente (OR = 0.5) y oriente (OR = 0.2).

**CONCLUSIONES:**

La baja frecuencia del registro de recomendaciones de actividad física encontrada, genera la necesidad de reforzar las estrategias de gestión de los centros de salud y fortalecer el monitoreo y acompañamiento en el cumplimiento de los protocolos de atención. Adicionalmente, es necesario capacitar a los equipos de salud sobre los beneficios de la actividad física y su prescripción adecuada, atendiendo a los múltiples beneficios derivados de su práctica sobre la salud materno-fetal.

## INTRODUCTION

Prenatal care is defined as the care provided, in general by health professionals, to all pregnant women to ensure the best health conditions for the woman and her fetus^1^. As in pregnancy women feel motivated to improve their habits and lifestyles to benefit their health and their child’s, the recommendations that health personnel provide to pregnant women are important^2^. Advice on healthy habits includes proper diet, physical activity (PA) practice and the appropriate weight gain during pregnancy^3^.

Scientific evidence shows that the practice of PA in pregnant women is associated with a lower probability of developing hypertensive alterations in pregnancy^4^, diabetes mellitus^5^, preterm delivery^6^ and cesarean section^7^. In addition, it contributes to a better control of weight gain during pregnancy^6^ and brings benefits to maternal mental health, decreasing the possibility of anxiety^8^ and depressive symptoms^9^. Therefore, it is necessary for health professionals to recommend and educate pregnant women about the regular practice of PA.

Despite the importance of PA for maternal-fetal health, a large part of the health staff does not offer recommendations on its practice and, in addition, its duration, frequency and intensity is not clear^10^. On the other hand, absolute rest is recommended, even in situations not clinically indicated^11^, despite the harmful effects it may have on the health of the mother-child binomial^12^.

Additional factors of cultural nature also have a negative influence on the practice of PA during pregnancy, among them the fear of harming the fetus or inducing an abortion, beliefs that have been transmitted and perpetuated by family and friends^10^ ^,^ ^13^. These factors, added to the lack of clarity by health personnel when offering recommendations, represent one of the most significant barriers to PA practice during pregnancy^14^.

Studies through maternal interviews indicate that between 39% and 55% of women do not receive education about their health care^15^ ^,^ ^16^. Other studies have focused on establishing the frequency of PA and whether this practice is based on a recommendation from health personnel^14^ ^,^ ^17^. However, few studies have explored how much prenatal care providers agreed with PA recommendations^18^. Few studies have quantified the record in the clinical history of PA and rest recommendations by health personnel during prenatal care; Yeoh, in a study from Malasya, found that this record existed in only 3.3% of medical records^16^.

On the other hand, in the literature reviewed only two studies reported the frequency with which pregnant women discussed with the health personnel in the prenatal control programs (PNCP) about rest. White et al. found that 76.9% of 1265 pregnant women interviewed by telephone reported having talked to health personnel about the need for rest during pregnancy^15^. On the other hand, Yeoh et al.^16^, in their study based on the review of medical records, only registered the average number of times that pregnant women received recommendations, finding that this was a regular practice in PNCP, with an average of 2.7, against the average of 0.03 times of physical activity recommendations.

In the literature reviewed in Colombia, only two studies evaluated compliance with the activities carried out in the PCPN^19^ ^,^ ^20^; Arias et al.^19^ found that more than 50% of the pregnant women interviewed received PA recommendations from the nursing staff; and Álvarez et al.^20^, although they evaluated that the health team agreed with the PNCP protocol by reviewing medical records and interviewing users, they did not measure how much they agreed with the provision of PA and rest recommendations from the clinical record. To know the frequency of PA and rest recommendations registry will contribute to the decision making by the directors of the PNCP aimed at reinforcing the PA counseling, identifying the areas that require interventions; therefore, this work aimed to determine the frequency of the PA and rest recommendations registry in the pregnant women’s medical records and to explore their associated factors in a primary care level PNCP in Bucaramanga, Colombia.

## METHODS

### Context of the Research

The study was carried out in Bucaramanga, capital of the department of Santander (Colombia). This city is nationally recognized because since 2009 it has been implementing strategies with a Primary Health Care (PHC) approach that transcend those stipulated by the General System of Social Security in Health.

The municipal health authority is the Secretary of Health and Environment, which – through the Social Enterprise of the State Institute of Health of Bucaramanga (ESE-ISABU) – provides primary health care services for low-income population served in 24 urban health centers (HC), two rural mobile HCs, an urban extramural team, a Maternal and Child Intermediate Health Unit (UIMIST) and a Local Hospital (LH).

Bucaramanga is divided into 17 communes, defined as administrative subdivisions made up of neighborhoods, settlements, urbanizations and other sectors with a floating population^21^. The HCs offer their services in 12 of the 17 communes of the city, which corresponds to 45% of the total population, characterized by being poor, with health insurance coverage via State subsidies. For their administration and supervision, the HCs are organized by administrative zones: north, east, west and south, with the northern zone being the most socioeconomically depressed; each area has an assigned coordinator, who manages the programs that the HCs provide.

ESE-ISABU offers low-risk PNCP in HCs. For their operation, they have health professionals. All pregnant women are evaluated by medical, nursing and dentistry professionals; attention for nutrition and psychology is offered according to the particular needs of each pregnant woman and based on the referral made by medical and nursing professionals. When women in their first control or in a subsequent control are classified as high risk, they are referred to the UIMIST or the LH; if the problem is solved, they are sent back to the respective HC so that they can continue their control.

All HCs follow the guidelines of Resolution 412 of the year 2000, specified in the National Technical Standard for the Detection of Pregnancy Disorders^22^, and those of the low-risk prenatal care protocol, typical of the ESE-ISABU, which contains activities complementary to those proposed by the national standard. Both Resolution 412 and the ESE-ISABU protocol recommend carrying out individual education for pregnant women on PA; however, neither of these two documents specifies the content of these recommendations in terms of frequency, intensity and duration of the PA.

It is necessary to establish the differences between a recommendation, a prescription and an education about human behavior in a control program. The first is related to counseling on the importance of performing PA; the second specifically contains the type of PA and the domains of duration, frequency and intensity, in order to improve health condition; finally, the third is related to a bidirectional and interactive process of behavioral change, from learning and making decisions aimed at improving health and well-being, in which the health personnel must ensure the understanding of information about which one is being educated^23^.

### Source of Information for Analysis

A secondary analysis of the information collected in the project called “Technical efficiency of health promotion and disease prevention programs for women in the public primary care network of Bucaramanga, Colombia” was carried out. The methodology of this project is widely described in Ruiz-Rodríguez et al.^24^

The sampling frame consisted of the medical records of all pregnant women who attended at least one Prenatal Control (PNC) between January 1 and December 31, 2012 (n = 2,932) in 21 HCs of public first-level care, belonging to the ESE-ISABU. A random sample was selected between 16 and 60 clinical records in each HC, proportional to the total of pregnant women attended in each HC during 2012.

The research team, with the support of nutrition, nursing and medical professionals with experience in PNCP, generated a checklist to review the medical records. The checklist was adjusted after a pilot test with all the items included in the clinical record defined by the ESE-ISABU for the PNCP. The staff that supported the research team in the preparation of the checklist and pilot test took care of its completion, after training and standardization of procedures. In the case of PA and rest, the information of each PNC was carefully reviewed in the diagnostic and care plan sections.

### Variables

The presence of the record of PA and rest recommendation by medical, nursing and nutrition professionals are the dependent variables; these variables were dichotomized as yes or no.

The independent variables of the pregnant women, registered in the clinical record and included for the analysis, were:

- Sociodemographic: age (years old completed), education (last school year approved), economically remunerated occupation (yes, no), marital status, according to cohabitation with partner (yes, no) and socioeconomic stratum (1, 2 and ≥ 3, 1 being the lowest).
- Anthropometric measurements: pre-gestational body mass index (BMI) categorized as low weight, normal weight and excess weight, taking as reference the cut-off points of the World Health Organization (WHO).
- Background: nulliparity (yes, no), presence of at least one obstetric history such as diabetes, pregnancy-induced hypertension, preeclampsia, eclampsia or kidney disease in previous pregnancies (yes, no).
- Related to the PNCP: gestational age at the beginning of the PNCP (trimester), high or medium obstetric risk at the first PNC (yes, no), number of PNC performed (1–4 or > 4), diagnosis of at least one health condition such as anemia, threatened abortion, placenta previa or intrauterine growth restriction, according to laboratory results and ultrasound in at least one NPC (yes, no).
- Administrative service area (north, west, east and south).

### Analysis

A descriptive analysis of the pregnant women characteristics was carried out through measures of central tendency and dispersion for the continuous variables and frequency tables for the categorical variables. The bivariate analysis was carried out among the dependent variables, with each of the continuous independent variables using the Mann-Whitney U test, and in the case of the categorical ones the chi-squared test was applied.

To estimate the factors associated with PA recommendation, a crude and adjusted analysis was made between the independent variables with the PA recommendation record, applying a logistic regression model following the Greenland^25^ recommendations to estimate the odds ratio and 95%CI; this model was adjusted for pre-pregnancy BMI, nulliparity, number of PNC performed, diagnosis of high or medium obstetric risk in the first PNC and administrative care area.

Given the low frequency of rest recommendations, it was not possible to elaborate a multivariate model, so only associations of bivariate analysis are shown. All the analyzes were carried out in Stata 14.1 with a level of significance α = 0.10, given the exploratory nature of this work.

The study was endorsed by the ethics committee of the Bucaramanga Health Institute.

## RESULTS

### General Description

We reviewed the medical records of 838 pregnant women between 13 and 46 years old who attended at least one PNC between January 1 and December 31, 2012 at 21 HCs of Bucaramanga. The medians of age and schooling years were 22 years (RIC = 19–27) and nine years (RIC = 6–11) respectively; 60.5% of pregnant women were nulliparous, 59.5% started PNC in the first gestational trimester and 56.6% were classified as high or medium obstetric risk during the first PNC (Table 1).

**Table 1:** Sociodemographic, anthropometric characteristics and antecedents registered in the clinical records of pregnant women attending prenatal control programs in Bucaramanga, Colombia. (n = 838)

Characteristics	n	%
Sociodemographic		

Age (years old) Median (RIC)	22	19–27
Education (years) Median (RIC)	9	6–11
Paid occupation	270	32.3
Live with partner	637	77.6
Socioeconomic stratum		
1	329	39.7
2	309	37.3
> 3	190	22.9

Anthropometry		

BMI (first PNC)		
Under Weight	65	8.0
Normal weight	476	58.3
Overweight	275	33.7

Background		

Nulliparous	505	60.5
Obstetric history^a^	353	42.2

Related to the PNCP		

Gestational trimester (first PNC)		
1	465	59.5
2	227	29.0
3	90	11.5
Obstetric risk (first PNC)^b^	427	56.6
More than 4 PNCs made	457	54.5
Diagnostics during PNC^a,c^	128	15.3

Administrative area of the care center		

North	250	29.8
West	229	27.3
East	166	19.8
South	193	23.0

RIC: interquartile range; BMI: body mass index; PNC: prenatal control; PNCP: Prenatal Control Program

^a^ Includes diagnosis of diabetes, pregnancy-induced hypertension, preeclampsia, eclampsia or kidney disease in previous pregnancies.

^b^ Includes high and medium obstetric risk.

^c^ Includes diagnoses of anemia, threatened abortion, placenta previa or intrauterine growth restriction in at least one prenatal check-up.

### Recommendations of PA

PA recommendation was found in 26.1% of pregnant women’s medical records; however, its type, time, frequency or duration was not specified. This registry was made mainly by nutrition professionals (97.3%), compared with medical (2.7%); it is emphasized that the nursing staff did not make PA recommendations.

In the bivariate analysis of factors associated with PA recommendation record (Table 2), it was found that pregnant women belonging to the lowest socioeconomic stratum, known in Colombia as stratum 1, with normal pregestational weight, nulliparous, in the first gestational trimester at the time of the first PNC, attended more than four PNCs, that were not diagnosed as high or medium obstetric risk in the first PNC, and that performed PNCs in northern zone institutions were more likely to register a PA recommendation in their medical record (Table 2).

**Table 2:** Sociodemographic, anthropometric, clinical characteristics and personal history of pregnant women, according to the registry of physical activity and rest recommendation in the clinical record.

Characteristic	Registry of PA recommendations				p	Registry of rest recommendations				p
	
	Yes (n = 219)		No (n = 619)			Yes (n = 30)		No (n = 808)		
			
	n	%	n	%		n	%	n	%	
Characteristics										

Age (years old) Median (RIC)	22	18–26	23	19–27	0.037	25	21–27	22	19–27	0.051
Education (years) Median (RIC)	10	6–11	9	6–11	0.507	7.5	5–11	9	6–11	0.160
Paid occupation	66	30.1	204	33.1	0.426	8	26.7	262	32.5	0.502
Live with partner	171	79.5	466	76.9	0.426	26	92.9	611	77.0	0.049
Socioeconomic stratum										
1	97	44.7	232	38.0	0.004	14	46.7	315	39.5	0.095
2	88	40.5	221	36.2		14	46.7	295	37.0	
> 3	32	14.7	158	25.9		2	6.7	188	23.6	

Anthropometry										

BMI (first PNC)										
Under Weight	14	6.4	51	8.5	0.020	2	6.7	63	8.0	0.850
Normal weight	144	66.4	332	55.4		19	63.3	457	58.1	
Overweight	59	27.2	216	36.1		9	30.0	266	33.8	
Medical History										
Nulliparous	148	67.6	357	57.9	0.012	10	33.3	495	61.5	0.002
Obstetric history^a^	89	40.6	264	42.8	0.580	13	43.3	340	42.2	0.900

Related to the PNCP										

Gestational trimester (first PNC)										
1	135	66.2	330	57.1	0.032	25	86.2	440	58.4	0.009
2	54	26.5	173	29.9		4	13.8	223	29.6	
3	15	7.3	75	13.0		0	0	90	11.9	
Obstetric risk (first PNC)^b^	78	40.4	349	62.1	< 0.001	15	62.5	412	56.4	0.551
More than 4 PNCs made	157	71.7	300	48.5	< 0.001	24	80.0	433	53.6	0.004
Diagnostics during PNC^c^	33	15.1	95	15.3	0.921	3	10.0	125	15.5	0.413

Administrative area of the care center										

North	100	45.7	150	24.2	< 0.001	11	36.7	239	29.6	0.491
West	51	23.3	178	28.8		6	20.0	223	27.6	
East	13	5.9	153	24,7		4	13.3	162	20.0	
South	55	25.1	138	22.3		9	30.0	184	22.8	

RIC: interquartile range; BMI: body mass index; PNC: prenatal control; PNCP: Prenatal Control Program

The differences in the PA and rest recommendations according to the independent variables were estimated by the Mann-Whitney U test for age and education and with the chi-square test for the other variables.

^a^ Includes diagnosis of diabetes, pregnancy-induced hypertension, preeclampsia, eclampsia or kidney disease in previous pregnancies.

^b^ Includes high and medium obstetric risk.

^c^ Includes diagnoses of anemia, threatened abortion, placenta previa or intrauterine growth restriction in at least one prenatal check-up.

The factors analyzed in the multivariate model showed positive and statistically significant associations for nulliparous pregnant women (OR = 1.7 95%CI 1.1–2.4) and for those who attended more than four PNC (OR = 2.2 95%CI 1.5–3.2); in contrast, negative and statistically significant associations were found between the PA recommendations and pregnant women with high or medium obstetric risk in the first PNC (OR = 0.6 95%CI 0.4–0.9) and those that were attended in the HC of the west (OR = 0.5 95%CI 0.3–0.8) and east (OR = 0.2 95%CI 0.1–0.3) administrative areas (Table 3).

**Table 3:** Factors associated with the registration of physical activity recommendations to pregnant women attending prenatal care programs in Bucaramanga (Linktest p = 0.266).

Characteristic	Not Adjusted			Adjusted		
	
	OR	95%CI	p	OR	95%CI	p
BMI (first PNC)						
Under Weight	0.6	0.3–1.2	0.15	0.6	0.3–1.2	0.138
Normal weight	Ref.			Ref.		
Overweight	0.6	0.4–0.9	0.009	0.7	0.5–1.1	0.103
Nulliparous						
No	Ref.			Ref.		
Yes	1.5	1.1–2.1	0.013	1.7	1.1–2.4	0.007
Obstetric risk (first PNC)ᵇ						
No	Ref.			Ref.		
Yes	0.4	0.3–0.6	< 0.001	0.6	0.4–0.9	0.010
PNC number						
1–4	Ref.			Ref.		
> 4	2.7	1.9–3.7	< 0.001	2.2	1.5–3.2	< 0.001
Administrative area of the care center						
North	Ref.			Ref.		
West	0.4	0.3–0.6	< 0.001	0.5	0.3–0.8	0.008
East	0.1	0.1–0.2	< 0.001	0.2	0.1–0.3	< 0.001
South	0.6	0.4–0.9	0.012	0.8	0.5–1.3	0.459

BMI: body mass index; PNC: prenatal control.

### Recommendations of Rest

Only 3.6% of PNCP users registered rest recommendations in the clinical record, with physicians prescribing it more frequently (86.7%), comparing with nurses (13.3%). Additionally, it was evidenced that pregnant women who lived with a partner, were multiparous, in the first gestational trimester at the time of the first PNC and attending later to more than four PNCs were more frequently registered with rest recommendations (p < 0.05) (Table 2).

## DISCUSSION

The objective of this secondary analysis was to establish the frequency and possible factors associated with the registry of PA and rest recommendations in the clinical record of PNCP pregnant women in a Colombian city. Its importance lies in the fact that it is the first time that data are presented from the pregnant women’s medical records, unlike other studies, which were based on interviews with women^19^ ^,^ ^20^. Although the information from clinical records has limitations, these are less than those that are derived from studies from surveys with long-term reminders, given their potential information bias.

Our results show that 26.1% of pregnant women had a record of PA recommendations in PNCP; this frequency is greater than 3.3% reported by Yeoh et al.^16^; the differences can be explained by the type of record used in each study.

In the multivariate analysis, no sociodemographic characteristics of the pregnant women were associated with the registration of PA recommendations. On the other hand, nulliparity, the obstetric risk at the beginning of the PNC, the PNC number and the administrative area were associated. Having children was negatively associated with registering the PA recommendation during the PNC consultation. These findings coincide with that reported by Santo et al.^17^, who found that a high parity was significantly associated with PA recommendations by health personnel. To the date, a negative association between equality and PA practice during pregnancy has been documented, attributing this situation to the lack of time of some pregnant women, because they must take care of their children at home^26^.

In the sample analyzed, it was found that pregnant women who attended between one and four PNCs were rarely recommended on PA, compared with those who attended more than four PNCs; This may be because those who attended more than four PNCs were closer to the health personnel and received more detailed follow-up of their pregnancy, facilitating the counseling of the pregnant women in different aspects, including PA; however, in the literature reviewed there was no evidence to support this finding.

Our results showed a negative association between high or medium obstetric risk during the first PNC and the record of PA recommendations in the clinical record; this situation may be due to the abstention of health personnel to recommend the PA practice in this group of pregnant women for fear of harming the mother’s or child’s health^13^.

Additionally, we found statistically significant associations between the administrative area to which the HC belonged and the registry of PA recommendation; this probably shows differences in the administration of the HC in each zone and not in the conglomerate of socioeconomic strata that conform them, which is evidenced in the bivariate analysis results, considering that if it represented the socioeconomic stratum of the geographic zones, a significant effect of this variable would have also been found.

It has been found that during PNC consultations other aspects related to the health of pregnant women are prioritized and PA is relegated to the background^19^ ^,^ ^27^. Additionally, some authors have reported that one of the main barriers in prescribing PA is the lack of information by health professionals about international recommendations for safe PA practice^14^ ^,^ ^27^. In this sense, a qualitative study in pregnant Latinas residing in the United States showed that the participants expressed a certain degree of confusion by the health personnel when offering the PA counseling^10^. Contrary to what was observed in the PA prescription during pregnancy, the American College of Obstetricians and Gynecologists (ACOG) has warned about the high prescription of rest during pregnancy, even without being really indicated, despite its harmful effects on maternal-fetal health^11^ ^,^ ^28^.

Because of the importance of the role played by health personnel in maternal-fetal well-being, our findings pose challenges for the local health system. On the one hand, the need to reinforce HC management strategies and the strengthening of monitoring and accompaniment in compliance with care protocols; on the other hand, the need to train their health teams regarding the prescription of PA for pregnant women, so that they are aware that PA and rest are individual education interventions of the PNC consultation^15^ ^,^ ^17^ ^,^ ^29^. The fact that no detailed records on the PA recommendations were found in the medical records indicates that there is a lack of knowledge on how to make the prescription of PA for pregnant women. This situation also raises the need to formulate specific guides on pregnant PA in the framework of the PNCP. Likewise, the results of this study suggest the need for higher education institutions to include in their curricula PA and rest recommendations as an aspect of comprehensive care for pregnant women within the framework of the maternal and perinatal *Ruta Integral de Atención en Salud* (RIAS) that will begin to be implemented this year.

Contrary to the aforementioned studies, our results showed that only 3.6% of pregnant women were recommended for rest. These differences can be explained by a possible underreporting of these recommendations by PNC professionals.

One of the main limitations of this study and, in particular, of the secondary analyzes derived from clinical histories, is that there is a potential underreporting of information, which would affect the frequencies of record detected and the potential associations to be evaluated. However, the random selection of the clinical histories analyzed during the year of analysis, as well as the sampling frame, makes it possible to generalize the findings.

On the other hand, more studies documenting the figures and factors associated with PA and rest recommendation in PNCP are needed to facilitate discussion and comparisons, in order to propose new strategies in Latin American countries to overcome the difficulties registered here.

## CONCLUSIONS

In summary, a low frequency was found in the registry of PA recommendations and an even lower registry of rest recommendations during prenatal care in a socioeconomic vulnerable population. These findings suggest that being the PNCP a scenario with an enormous potential to provide counseling and recommendations on healthy practices, the users of the PNCP of the study did not have the opportunity to receive information or advice on practices regarding PA and rest, which could deepen even more their social vulnerability level. On the other hand, the preponderant role of nutrition professionals in the registry of recommendations, comparing with those of medical and nursing areas, is highlighted and also poses challenges in terms of training on PA and rest recommendation.

The results of this study are also a baseline for proposing strategies that increase both the quantity and quality of PA and rest recommendations, which will contribute to achieve health outcomes in maternal and perinatal RIAS within the framework of the new Comprehensive Health Care Policy (PAIS) promulgated in 2016.
